# Psychometric Validation of the Macedonian-Language Version of the Ultrashort Five-Item Oral Health Impact Profile in the North Macedonian Population (OHIP5-MAC)

**DOI:** 10.3390/medicina61040655

**Published:** 2025-04-02

**Authors:** Saša Elenčevski, Asja Čelebić, Aleksandra Popovac, Sonja Apostolska, Julijana Nikolovska, Ivica Stančić

**Affiliations:** 1 Department of Prosthodontics, Faculty of Dentistry, Ss. Cyril and Methodius University of Skopje, 1000 Skopje, North Macedonia; selencevski@stomfak.ukim.edu.mk (S.E.); jnikolovska@stomfak.ukim.edu.mk (J.N.); 2 Department of Removable Prosthodontics, School of Dental Medicine, University of Zagreb, 10000 Zagreb, Croatia; 3 Department of Prosthodontics, School of Dental Medicine, University of Belgrade, 11000 Belgrade, Serbia; aleksandra.popovac@stomf.bg.ac.rs (A.P.); ivica.stancic@gmail.com (I.S.); 4 Department of Tooth Pathology and Endodontics, Ss. Cyril and Methodius University of Skopje, 1000 Skopje, North Macedonia; sapostolska@stomfak.ukim.edu.mk

**Keywords:** ultrashort questionnaire, OHIP-5, oral health-related quality of life, reliability, validity, responsiveness, Macedonian language, North Macedonia

## Abstract

*Background and Objectives*: Due to a lack of an ultrashort questionnaire for Oral Health-Related Quality of Life (OHRQoL) assessment in the North Macedonian population, the OHIP5 was translated into Macedonian language with aim to test its psychometrical properties. *Materials and Methods*: Two types of reliability were tested: internal consistency by calculating Cronbach’s alpha coefficient (general population), and test-retest reliability by calculating intraclass correlation coefficients (ICC) in a convenient sample of dental students who answered the same questions twice. Two types of validity were also tested: convergent validity (via a Spearman rank correlation) and known-group validity (via a Mann–Whitney U test). Responsiveness was tested by calculating the significance of the differences between the pre-treatment and after-treatment scores and by calculating the effect sizes of different dental treatments. *Results*: The Cronbach alpha coefficient of 0.756 and inter-item correlations above 0.2 pointed out good internal consistency. The test-retest reliability was confirmed by high ICCs and no significant differences between the OHIP5-MAC scores in a period of two weeks as dental students had no oral health changes. The Spearman rank correlation of −0.88 (*p* < 0.01) between the OHIP5-MAC summary scores and one simple question scoring self-perceived oral health (1–5; 1-worst oral health; 5-excellent oral health) confirmed the convergent validity. Significant differences between subjects with natural teeth and those with removable dentures (*p* < 0.01) confirmed the know-group validity, as it was predicted that removable denture wearers would have more impairment of oral health than individuals with natural teeth. The sensitivity of a questionnaire to measure changes elicited by dental treatments, i.e., responsiveness, was confirmed in individuals who received different dental treatments and scored significantly better (lower scores) their OHRQoL one month after the treatment, compared to their pretreatment scores (*p* < 0.05). *Conclusions*: Generally good psychometric properties of the OHIP5-MAC justify the recommendation for its future use in clinical settings and research.

## 1. Introduction

Assessing Oral Health-Related Quality of Life (OHRQoL) in large, diverse populations requires a simple, low—burden questionnaire for respondents. However, the instrument must be psychometrically validated to enable comparisons. The OHIP49 questionnaire, developed by Slade several decades ago, was considered to consist of seven domains [1], which is the same as the shortened OHIP14 version [2]. Empirically, the seven domains were as follows: Functional Limitation, Physical Pain, Psychological Discomfort, Physical Disability, Psychological Disability, Social Disability, and Handicap [1,2]. Due to the long duration required to fill in the questionnaire and consequent unanswered items, the shortened version, i.e., the OHIP14 questionnaire, has been developed and translated into many languages and validated in many countries worldwide [2,3,4,5,6,7,8,9,10,11,12,13,14,15,16,17,18,19,20,21,22,23,24,25,26,27,28,29,30,31,32,33,34].

However, using the large database collected from several countries in 2014 [35], the studies provided the exploratory and confirmatory factor analysis [36,37], which proved that the OHIP questionnaire consists, in fact, of only four oral health dimensions: Oral Function, Oro-facial Pain, Oro-facial Appearance, and Psychosocial Impact. The four-dimensional model was additionally confirmed in other multicenter studies [38]. To reduce the burden to both respondents and professionals, the ultra-short OHIP-5 questionnaire has also been developed with one item representing at least each of the four dimensions of oral health [36,37,39,40,41,42] and validated in several countries.

In the Macedonian language, the OHIP49 [43], OHIP-14 [21], and OHIP-EDENT [44] questionnaires have already been validated, but the ultra-short version of the OHIP questionnaire, i.e., the OHIP5, has not yet been psychometrically tested. To obtain an ultra-short questionnaire for the assessment of OHRQoL in the North Macedonian population, the aim of the study was to develop the OHIP5-MAC questionnaire and test its psychometric properties.

## 2. Materials and Methods

### 2.1. Translations of the OHIP5 Questionnaire into the Macedonian Language

The English version of the OHIP5 Questionnaire was translated into the Macedonian language using the forward-backward translation. As the first step, a professional translator, working together with a dentist who has an excellent knowledge of the English language after spending one year in an English language speaking country, translated the five items of the questionnaire into the Macedonian language. Then, four specialists, together with four patients (a specialist in Endodontics together with one patient, a specialist in Prosthodontics together with one patient, a specialist in Oral surgery together with one patient, and a specialist in Oral medicine together with one patient), reviewed the translation for clarity. When approved, the questionnaire was back-translated mutually by one Macedonian dentist who had been working for nine years in an English language speaking country and another professional translator. Finally, the back-translation was compared with the original English version by three native speakers of the English language. The translated North Macedonian OHIP5 questions (with English language version in parentheses) are presented in Appendix A.

### 2.2. Participants and Data Collection

Subjects who participated in the study were selected from the general population (*n* = 164), dental students (*n* = 48), and dental patients with different treatment needs (*n* = 39). Mean age, age range, gender, as well as research purposes are shown in Table 1. Participants in the general population represented mostly a random sample of subjects willing to participate, while subjects selected to assess test-retest reliability and responsiveness represented a convenience sample. Exclusion criteria in the dental student group were any noticeable changes or treatments in the oral cavity between the two observation periods. In the patient group, subjects who were unable to walk independently, who had malignant or systemic diseases, or who had a history of depression/anxiety or any disease that might prevent them from attending the follow-up examination or influence their opinion on the results of a therapy were excluded. The study was approved by the Institutional Ethics Committee (Faculty of Dentistry Skopje, St. Cyril and Methodius University, North Macedonia). All participants received the written information explaining the purposes of the study and voluntarily took part. A total of 164 individuals were selected from the general population, i.e., from subjects who came for a preventive blood pressure check and from subjects who came to see a theatre performance. All subjects filled in the OHIP5-MAC questionnaire. The responses to the items of the OHIP5-MAC were given on the five-point Likert scale, with scoring from zero to four (zero = never; one = hardly ever; two = occasionally; three = fairly often; and four = very often). The lower scores represented better oral health. They also graded their oral health on the Likert scale from one to five (higher grades representing better oral health). The respondents also gave information about wearing removable dentures (yes or no). All questions of the OHIP5-MAC questionnaire referred to the period of the previous seven days [45].

Dental students were a convenient sample and answered the five questions from the OHIP5-MAC questionnaire twice within a period of two weeks. Another convenience sample was selected among participants who needed different dental treatments. They underwent a clinical triage examination and, when necessary, a radiological examination. The dental treatments were made by a specialist in the respective dental fields (Table 1). To validate the responsiveness of the ultrashort questionnaire, the patients filled in the OHIP5-MAC questionnaire twice: before the treatment and one month after the treatment.

### 2.3. Statistical Analysis

#### 2.3.1. Reliability

An assessment of the reliability of the OHIP5-MAC was done using internal consistency and test-retest. The internal consistency was assessed from the data collected in the general population by calculating the Cronbach alpha coefficient and inter-item correlations. All values > 0.7 were considered acceptable, and inter-item correlations > 0.2 were also considered acceptable [46].

The test-retest reliability was tested on the convenience sample consisting of 48 dental students who answered the OHIP5-MAC questions twice within a period of two weeks without any changes in their oral status during that period. Based on the one-way repeated-measures analysis of variance (ANOVA), the intraclass correlation coefficients (ICC) were calculated [47].

#### 2.3.2. Validity

Convergent validity and known-groups validity were analyzed in the general population. Convergent validity was assessed from the association between scores of the reported self-perceived oral health and the OHIP5 summary scores by calculating the Spearman rank correlation. Known-groups validity was assessed by testing the significance of the differences between the OHIP5-MAC summary scores of different groups expected to have different OHRQoL impairment, i.e., between participants having their natural teeth (or fixed partial dentures) and those wearing removable dentures. It was expected that participants wearing removable dentures would have more OHRQoL impairments than participants who had their own teeth, i.e., that removable denture wearers would have higher OHIP5-MAC summary scores. The Mann–Whitney test was used to test the significance of the differences between the groups, and the difference was considered significant when the *p* value was <0.05.

#### 2.3.3. Responsiveness

It was tested in a convenience sample of 39 dental patients who needed treatment and were referred to the specialist after a thorough examination. A total of 12 participants needed root canal endodontic treatment either due to acute (5 participants) or chronic pulpitis (7 participants). The diagnosis had to be confirmed radiologically, as well as the quality of root canal filling after the treatment. Fifteen patients needed complete dentures, four of them for the first time as they were edentulous during the period of wound healing after the last teeth extractions. Seven patients, already complete denture wearers, received two implants in the mandible, and after the period of osseointegration, new maxillary complete dentures and new mandibular implant-supported overdentures were made and delivered. Five patients received lithium-disilicate crowns and/or veneers in the maxillary anterior region for aesthetic reasons (Table 1). It was predicted that OHRQoL impairment would be reduced one month after the treatment when the participants would fill in the OHIP5-MAC questionnaire for the second time. The first time the OHIP5-MAC was filled in was before any treatment. The standardized effect size was calculated [48]. The effect size of 0.2 was considered small, 0.5 medium, and 0.8 large. The effect size > 0.5 was considered clinically significant [49].

## 3. Results

### 3.1. Reliability

#### 3.1.1. Internal Consistency

Mean values of each OHIP5-MAC item, standard deviations, and inter-item correlations are presented in Table 2. The Cronbach alpha coefficient was 0.756. It presented sufficient covariance among the items indicating good reliability of the questionnaire. When one item was deleted, the Cronbach alpha coefficient ranged from 0.66 to 0.77. Inter-item correlations of the OHIP5-MAC instrument ranging from 0.21 to 0.68 are presented in Table 3, and were also satisfactory, as the values were above 0.20.

#### 3.1.2. Test-Retest Reliability

Another measure of reliability, i.e., the stability of the results when no changes occurred in the OHRQoL, was also tested. For that purpose, 48 dental students participated and filled in the same OHIP5-MAC questionnaire within a period of 14 days. The results are presented in Table 4. All ICC values were acceptable, and there were no significant differences between the results of the first and the second time. The OHIP5-MAC scores did not change significantly without changes in the respondents’ OHRQoL (*p* > 0.05). The item: “Difficulty doing usual jobs” was not computed, as there were no differences at all in a period of 14 days, i.e., the difference equaled zero.

### 3.2. Validity

#### 3.2.1. Convergent Validity

Convergent validity was assessed using the association between the reported self-perceived oral health (Likert scale from one to five, with higher scores representing better oral health) and the OHIP5 summary scores (Likert scale from zero to four, with higher scores representing worse oral health) by calculating the Spearman rank correlation. The coefficient was −0.88 (*p* < 0.01). Sixty-five participants assessed their oral health as excellent and had mean OHIP5-MAC summary scores of 0.38 and a standard deviation (SD) of 0.8. Sixty-seven participants from the general population assessed their oral health as four. Their mean OHIP5-MAC Summary score was 3.6 and SD was 1.9. Twenty-nine subjects assessed their oral health as three and had a mean OHIP5-MAC score of 7.56 with 1.81 SD. Eleven subjects had an oral health score of two and had mean OHIP5-MAC summary scores of 11.67 with SD 1.15. None of the participants gave the worst score (one) to her/his oral health.

#### 3.2.2. Known-Group Validity

Known-group validity, also known as divergent validity, assessed the differences between the OHIP5-MAC score of each item as well as the summary scores of different groups which were expected to have different OHRQoL impairment (subjects with natural teeth were compared with those wearing removable dentures). Known-group validity was confirmed by the results of the Mann–Whitney test. Each of the items, as well as the OHIP5-MAC summary scores, were significantly higher (more impairment) in removable denture wearers (Table 5) than in participants with their own teeth (or fixed-partial dentures, i.e., crowns and bridges), as predicted.

### 3.3. Responsiveness

To assess whether the summary score or each item of the OHIP5-MAC is sensitive to change elicited by a treatment (which was done by a specialist in the respective field of dentistry), the responsiveness was tested using the Wilcoxson Rank Signed test and by calculating the standardized effect size. The scores of all 39 patients together, as well as scores of the patients with specific treatment needs, are shown in Table 6. Only items with significant differences between the pretreatment scores and the post-treatment scores are shown in the table. All items and the summary scores of all 39 patients together were significantly reduced after the treatment with large effect sizes, except for the item: “Less flavor in food”, which showed a small/medium effect size. Endodontic patients did not present significant differences for items: “Uncomfortable about appearance” and “Less flavor in food”, but all other items had large effect sizes and the overall OHRQoL improved significantly after the treatment. In patients who received new complete dentures, all items showed large effects except the item: “Difficulty doing usual jobs”, which also changed significantly, but had a small effect size. Patients who received mandibular implant-supported overdentures had large effect sizes of all items except for the item: “Less flavor in food”, which showed no significant difference after the treatment. Patients who received lithium-disilicate crowns or veneers in the anterior maxillary regions had large effect sizes only for those items related to aesthetics and for the item difficulty doing usual jobs. However, the OHIP5-MAC summary score also changed significantly (*p* < 0.05).

## 4. Discussion

To minimize burdens and unanswered questions and improve data collection in interviews and phone calls, the ultra-short OHIP questionnaire, consisting of five items, was developed [39,40,41,42,50]. It has been translated into many languages and validated in different cultural environments [27,39,40,41,50,51,52,53,54,55,56].

Partial and complete edentulism are highly prevalent in North Macedonia and the Western Balkans, along with the widespread need for dental treatment. OHRQoL and prosthodontic treatment outcomes are influenced by ethnicity and place of residence (rural or urban). Economic transition and limited public dental care further complicate the situation and require oral health improvement programs [57,58]. These programs that are planned or have already started in North Macedonia must be evaluated using reliable instruments. Shorter and more effective tools are preferred, such as the OHIP5. Moreover, the instrument may be suitable for comparative analysis between Western Balkan countries due to similarities in language, cultural environment, and oral health experience.

Marginalized populations may perceive oral health as less important despite its significance in quality of life. This supports the recommendation to use the ultra-short questionnaire in large-scale, international and multi-center studies [40,41]. In North Macedonia’s multicultural population, the high demand for dental treatment highlights the need for such a questionnaire to assess treatment needs and options [58]. Therefore, this study aimed to validate the OHIP5 instrument. The translation was done using the accepted forward-backward methods. The results revealed good psychometric properties of the OHIP5-MAC.

The age of the participating subjects from the general population was similar to other OHIP5 validation studies, as well as the higher percentage of women [39,50,52,53,54,55,56].

Any questionnaire can be considered reliable when it gives the same result when repeated without changes of content and when the items are closely related. Reliability testing was done by two measures of reliability, i.e., internal consistency and test-retest reliability. Internal consistency is a measure based on the correlations of the items. The calculated Cronbach’s α coefficient of 0.756 was satisfactory, although each of the five items of the questionnaire referred to one of the four dimensions of oral health [35,36,37,38]. However, the coefficient was lower than in other OHIP-MAC versions (OHIP49, OHIP14, and OHIP-EDENT, respectively) [21,43,44], or OHIP versions with 14 items from the neighboring Balkan countries (which could be reasonably expected due to the reduced number of items in the ultrashort version) [9,11,31,32,59]. The Cronbach alpha coefficient was similar to Swedish, German, English, Persian and Arabic OHIP5 versions [27,39,50,52,54], while the coefficient was higher in Japanese (0.81) [56] and Spanish (0.83) versions [53]. Additionally, the corrected item correlations were above 0.2 in the OHIP5-MAC, which was considered acceptable. The test-retest reliability was confirmed by the stability of the results (no significant differences when the same questionnaire was answered on two separate occasions without changes in oral health between the sessions), and by high ICCs.

The convergent validity of the OHIP5-MAC was evaluated using the criteria of “self-reported oral health”, similar to some other studies [27,39,50,56]. The Persian version used the three criteria: “self-reported oral health”, “self-judgment of the need for dental treatments”, and “the number of natural teeth in the oral cavity” [54]. Arabic version correlated the OHIP5 scores with both self-reported oral and general health [52]. The convergent validity was confirmed by a high and negative significant Spearman rank correlation coefficient of the OHIP5-MAC and a simple question of similar construct. The self-reported question on oral health status gave higher scores to better self-perceived oral health, while the OHIP5-MAC gave zero to no problems at all (better oral health), while higher scores represented worse oral health.

The OHIP5-MAC also distinctly differentiated between two groups that were predicted to have differences, i.e., between subjects with natural teeth and with removable dentures. It was predicted that removable denture wearers would have more problems and worse oral health than subjects with natural teeth or fixed partial dentures. It is known from the dental literature that removable denture wearers have more complaints, more sore spots, lower chewing forces, and more psycho-social impairment (as they feel insecure with their dentures) in comparison to subjects with natural teeth [60,61,62,63]. That is particularly emphasized in removable denture wearers having advanced alveolar bone atrophy due to insufficient removable denture stability [64,65,66]. As predicted (known-groups validity), removable denture wearers scored significantly higher scores of all items, as well as the OHIP5-MAC summary score (worse oral health) than subjects with their own teeth or fixed partial dentures. The German version correlated TMD pain, burning mouth sensations and self-reported halitosis with the OHIP5 scores, predicting that subjects with no TMD pain, no burning mouth sensations and less frequently reported bad breath would have lower OHIP5 scores [39]. The Arabic version included the potentially different groups (missing teeth, location of the missing teeth, periodontal status) to assess the known-group validity [52]. The Chilean version utilized seven items in the short OHIP version and compared the OHIP scores with the presence of caries, need for complex periodontal treatment, prosthetic needs, and an age that was younger than 70 years in the validation process [55].

One of the very important characteristics of any questionnaire is its sensitivity to change, or its score change after completing different dental therapies. Therefore, we tested the responsiveness of the OHIP5-MAC questionnaire to several different dental treatments, as was done in the Arabic OHIP5 version [52]. For that purpose, convenient groups of patients (*n* = 39) were selected. In the twelve patients who needed root canal therapy, the OHIP summary scores significantly reduced one month after the therapy. That was confirmed by the radiological findings as successful. However, orofacial esthetics (uncomfortable with appearance) and the item “Less flavor in food” did not change significantly after the endodontic treatment, once again indicating that each item represented one of the four dimensions of oral health. In patients whose therapy was the manufacture of new complete dentures, all items changed significantly with the highest impact in improvement of orofacial aesthetics. Among those patients, four of them received their complete dentures for the first time and resolved esthetic problems of being fully edentulous, while all other complete denture patients had dentures which were more than five years old. It is well known that orofacial aesthetics in complete denture wearers decrease over time due to acrylic teeth wear and reduction of the vertical dimension of the lower third of the face aggravated by alveolar bone atrophy [60,63,64,65,66,67]. Moreover, the colors change over time, and different stains from food and beverages or from some disinfection agents used for oral hygiene maintenance are absorbed into the acrylic resin teeth and denture base due to PMMA porosity [68,69,70]. Therefore, it was not surprising to see the highest improvement in orofacial esthetics elicited by new complete denture delivery. The Japanese version also chose patients who needed complete dentures to test the responsiveness [56].

As many complete denture wearers have chewing difficulties and report pain originating from their denture-bearing area, mostly due to reduced stability of the mandibular complete denture in the atrophied mandible [65,66,67,68], seven patients whose therapy included mandibular two implant-supported new overdentures and new maxillary complete dentures were chosen. After implant insertion and the four-month period, the implants were opened and loaded by locator-type attachments in new overdentures. All item scores were reduced after the therapy, as expected, except for the item: “less flavor in food”, which did not change significantly, as patients still had full palatal coverage in the maxilla. The highest effect sizes registered for the items: “Difficulty chewing” and “Painful aching”, pointing out the sensitivity of change of specific items representing function (chewing) and orofacial pain, which was an expected result of the implant-supported overdenture therapy in the mandible [65,71,72,73,74,75,76].

Finally, in individuals having only aesthetic problems in frontal regions, the therapy with lithium-disilicate crowns or veneers significantly improved orofacial esthetics (item: “Uncomfortable with esthetics”) and psycho-social impact of impaired esthetics represented by the item: “Difficulty doing usual jobs”. The overall OHIP5-MAC summary scores were also reduced, with high effect sizes.

All assessments measuring responsiveness to a therapy were done one month after the treatment had been finalized, as it was predicted that pain due to endodontic treatment would reduce in that period [77,78,79], and that patients would adapt to their new dentures [68]. Moreover, all the patients were supposed to be more realistic about their esthetic and functional outcomes after some time than immediately after the therapy [78].

The new OHIP5-MAC questionnaire, due to its acceptable psychometric properties, can help establish an internationally comparable OHRQoL database for North Macedonia in future. Patient-reported oral health will be easily assessed using the OHIP5-MAC and compared with the results of other international researchers [41,80,81,82,83,84,85,86,87].

However, the study has some limitations. The information and all data were collected through self-reports in the general population; therefore, the statement of wearing removable dentures was not confirmed by clinical examinations. It is also possible that the subjects of the lowest income or education are missing from the available general population because subjects were selected among those who came for a preventive blood pressure check and from those who came to see a theatre performance. Moreover, very old subjects (over 80 years old), or/and those with severe oral or general health conditions are probably missing, as well as subjects from rural areas. Therefore, the generalization of the findings may be limited. The group of students (test-retest reliability; convenience sample) may be considered a consecutive or an availability sample, representing mostly individuals with healthy natural teeth, while other individuals of the same age, lower education, and different oral hygiene routines were omitted. Therefore, potential sampling bias cannot be completely ruled out. Additionally, only four different dental treatments were selected from the patient population to test the responsiveness to therapy. Not all treatments and treatment modalities were included, especially long-term ones, such as orthodontic treatments, which can last for several years. Future research would benefit from expanding the sample to more diverse populations.

## 5. Conclusions

Within the limitations of the study, the OHIP5-MAC questionnaire, which was psychometrically validated in the cultural environment of people living in North Macedonia and speaking the Macedonian language, showed good psychometric properties and, therefore, this ultrashort instrument can be recommended for clinical and research purposes, for future national epidemiological studies, and public oral health monitoring programs.

## Figures and Tables

**Table 1:** Mean age with standard deviations (SD), age range, gender and the reasons why participants took part in the OHIP5-MAC psychometric validation.

Sample	*n*	(% of Women)	Mean Age ± SD	Age Range (Years)	Research Purpose
General population	164	53%	42.30 (17.71)	18–76	Internal consistency, Concurrent validity, Known-group validity
Students	48	64.6%	23.56 (1.1)	21–25	Test-retest reliability
Patients with treatment needs:	39	59%	54.89 (16.37)	29–79	Responsiveness (sensitivity to change)
Endodontics	12	66.7%	40.1 (8.4)	29–53	
Complete dentures	15	46.7%	69.3 (6.6)	59–79	
Maxillary complete denture and mandibular implant-supported overdenture	7	71.4%	63.1 (8.23)	45–69	
Lithium-disilicate crown/crowns in maxillary anterior region	5	60.0%	35.6 (8.4)	29–50	

SD = standard deviation; *n* = number.

**Table 2:** Mean values of each OHIP5-MAC item, standard deviations, and coefficient alpha when an item was deleted.

OHIP5-MAC Item	Mean	Standard Deviation	Cronbach Alpha When Item Deleted
Difficulty chewing	0.87	0.98	0.66
Painful aching	0.59	0.71	0.71
Uncomfortable about appearance	0.84	1.07	0.70
Less flavor in food	0.83	1.20	0.77
Difficulty doing usual jobs	0.18	0.43	0.75

**Table 3:** Inter-item correlation matrix of the OHIP5-MAC.

Item of the OHIP5-MAC	Difficulty Chewing	Painful Aching	Uncomfortable About Appearance	Less Flavor in Food	Difficulty Doing Usual Jobs
Difficulty chewing	*	0.68	0.59	0.45	0.51
Painful aching	0.68	*	0.46	0.21	0.42
Uncomfortable about appearance	0.59	0.46	*	0.37	0.30
Less flavor in food	0.46	0.21	0.37	*	0.27
Difficulty doing usual jobs	0.51	0.42	0.30	0.27	*

* = not computed.

**Table 4:** Test-retest reliability, intraclass correlation coefficients, differences and significance between each OHIP5-MAC item and the summary score.

OHIP5-MAC Dental Students (*n* = 48)	ICC	Mean Difference (Standard Deviation)	95% Confidence Interval	*p*
Difficulty chewing	0.94	−0.02 (0.14)	−0.06–0.21	0.32 N.S.
Painful aching	0.88	0.06 (0.24)	−0.01–0.13	0.08 N.S.
Uncomfortable about appearance	0.93	0.06 (0.24)	−0.01–0.13	0.08 N.S.
Less flavor in food	0.70	−0.02 (0.14)	−0.06–0.21	0.32 N.S.
Difficulty doing usual jobs	*	*	*	*
Summary score	0.96	0.02 (0.25)	−0.05–0.09	0.57 N.S.

ICC = intraclass correlation coefficients.; * = not computed; N.S. = not significant

**Table 5:** Known-group validity for the OHIP5-MAC.

OHIP5-MAC	Removable Denture Wearers	N	Mean	Standard Deviation	Z	*p*
Difficulty chewing	no	105	0.34	0.58	−9.43	<0.001 **
	yes	59	1.80	0.85		
Painful aching	no	105	0.38	0.49	−4.41	<0.001 **
	yes	59	0.97	0.87		
Uncomfortable about appearance	no	105	0.47	0.87	−6.63	<0.001 **
	yes	59	1.50	0.78		
Less flavor in food	no	105	0.26	0.98	−10.23	<0.001 **
	yes	59	1.86	0.77		
Difficulty doing usual jobs	no	105	0.05	0.29	−4.70	<0.001 **
	yes	59	0.32	0.64		
OHIP5-MAC summary Score	no	105	1.47	1.99	−9.46	<0.001 **
	yes	59	6.47	2.34		

N = number of participants; Z = z value, *p* = significance of the differences; ** ≤0.01

**Table 6:** Responsiveness of the OHIP5-MAC by comparing pre-and post-treatment scores with standardized effect size.

OHIP5-MAC All Patients (*n* = 39)	Pre-Treatment x ± SD	Post-Treatment x ± SD	Z	*p*	Effect Size
Difficulty chewing	2.01 ± 1.14	0.59 ± 0.64	−4.93	<0.001 **	1.24
Painful aching	1.82 ± 1.26	0.33 ± 0.53	−4.87	<0.001 *	0.93
Uncomfortable about appearance	1.77 ± 1.22	0.26 ± 0.50	−4.68	<0.001 **	1.24
Less flavor in food	0.82 ± 0.82	0.49 ± 0.60	−3.36	0.001 **	0.40
Difficulty doing usual jobs	1.82 ± 1.29	0.05 ± 0.22	−4.92	<0.001 **	1.37
Summary score	8.23 ± 2.67	1.20 ± 1.34	−5.46	<0.001 **	2.63
**OHIP5-MAC** **Endodontic Patients (*n* = 12)**	**Pre-Treatment** **x ± SD**	**Post-Treatment** **x ± SD**	**Z**	***p***	**Effect Size**
Difficulty chewing	2.33 ± 0.65	0.47 ± 0.51	−3.13	0.002 **	2.86
Painful aching	3.17 ± 0.72	0.50 ± 0.52	−3.13	0.002 **	3.71
Difficulty doing usual jobs	2.25 ± 0.86	0.0 ± 0.0	−3.11	0.002 **	2.62
Summary score	8.83 ± 2.41	1.41 ± 1.08	−3.07	0.002 **	3.07
**OHIP5-MAC** **Patients: New Complete Dentures (*n* = 15)**	**Pre-Treatment** **x ± SD**	**Post-Treatment** **x ± SD**	**Z**	***p***	**Effect Size**
Difficulty chewing	2.40 ± 1.05	1.33 ± 0.52	−3.13	0.002 **	1.02
Painful aching	1.40 ± 0.74	0.47 ± 0.64	−3.12	0.002 **	1.26
Uncomfortable about appearance	2.20 ± 1.15	0.07 ± 0.26	−3.44	0.001 **	1.85
Less flavor in food	1.40 ± 0.82	0.49 ± 0.60	−2.12	0.034 *	1.19
Difficulty doing usual jobs	1.20 ± 1.57	0.13 ± 0.35	−2.41	0.016 *	0.68
Summary score	8.60 ± 3.35	2.80 ± 1.15	−3.42	0.001 **	1.73
**OHIP5-MAC** **Patients: Maxillary Complete Dentures and Mandibular Implant-Supported Overdenture (*n* = 7)**	**Pre-Treatment** **x ± SD**	**Post-Treatment** **x ± SD**	**Z**	***p***	**Effect Size**
Difficulty chewing	2.43 ± 1.13	0.14 ± 0.52	−2.07	0.023 *	2.03
Painful aching	1.71 ± 0.95	0.0	−2.26	0.024 *	1.80
Uncomfortable about appearance	1.29 ± 0.76	0.29 ± 0.49	−2.12	0.034 *	1.32
Difficulty doing usual jobs	1.57 ± 0.79	0	−2.41	0.016 *	1.28
Summary score	8.00 ± 1.23	1.00 ± 0.58	−2.39	0.017 *	5.69
**OHIP5-MAC** **Patients: Lithium Disilicate Crowns or Veneers in Anterior Maxilla (*n* = 5)**	**Pre-Treatment** **x ± SD**	**Post-Treatment** **x ± SD**	**Z**	***p***	**Effect Size**
Uncomfortable about appearance	3.20 ± 0.45	0.20 ± 0.45	−2.24	0.025 *	6.67
Difficulty doing usual jobs	3.00 ± 0.71	0	−2.06	0.039 *	4.20
Summary score	6.00 ± 1.41	0.2 ± 0.45	−2.06	0.039 *	4.11

*n* = number of patients, Z = Z value, *p* = significance of the differences, * ≤0.05; ** ≤0.01.

## Data Availability

The data presented in this study are available on request from the corresponding author. The data is not publicly available due to privacy.

## Appendix A

OHIP5-MAC QUESTIONS (Questions in the English language are in parentheses):

1. Дaли cтe имaлe пoтeшкoтии пpи џвaкaњe зapaди пpoблeми co вaшитe зaби, ycтa, пpoтeзи или вилици? (Have you had difficulty chewing any foods because of problems with your teeth, mouth, dentures, or jaw?)
2. Дaли cтe имaлe бoлки зapaди зaбитe или вo ycyaтa? (Have you had painful aching in your mouth?)
3. Дaли cтe ce чyвcтвyвaлe нeпpиjaтнo зapaди изглeдoт нa вaшитe зaби, зaбни пpoтeзи или вилици? (Have you felt uncomfortable about the appearance of your teeth, mouth, dentures, or jaws?)
4. Дaли cтe имaлe нaмaлeнo чyвcтвo зa вкyc зapaди пpoблeми co вaшитe зaби, ycтa, пpoтeзи или вилици? (Have you felt that there has been less flavor in your food because of problems with your teeth, mouth, dentures, or jaws?)
5. Дaли cтe имaлe пoтeшкoтии вo извpшyвaњeтo нa ceкojднeвнитe paбoти зapaди пpoблeми co вaшитe зaби, ycтa, пpoтeзи или вилици? (Have you had difficulty doing your usual jobs because of problems with your teeth, mouth, dentures, or jaws?)

## References

[1] Slade G.D., Spencer A.J. (1994). Development and evaluation of the Oral Health Impact Profile. Community Dent. Health.

[2] Slade G.D. (1997). Derivation and validation of a short-form oral health impact profile. Community Dent. Oral Epidemiol..

[3] Locker D., Allen P.F. (2002). Developing short-form measures of oral health-related quality of life. J. Public Health Dent..

[4] Osman S.M., Khalifa N., Alhajj M.N. (2018). Validation and comparison of the Arabic versions of GOHAI and OHIP-14 in patients with and without denture experience. BMC Oral Health.

[5] Soares G.H., Santiago P.H.R., Werneck R.I., Michel-Crosato E., Jamieson L.A. (2021). Psychometric Network Analysis of OHIP-14 Across Australian and Brazilian Populations. JDR Clin. Trans. Res..

[6] Ravaghi V., Farrahi-Avval N., Locker D., Underwood M. (2010). Validation of the Persian short version of the Oral Health Impact Profile (OHIP-14). Oral Health Prev. Dent..

[7] Motallebnejad M., Hadian H., Mehdizadeh S., Hajiahmadi M. (2011). Validity and reliability of the Persian version of the oral health impact profile (OHIP)-14. Casp. J. Intern. Med..

[8] Deshpande N.C., Nawathe A.A. (2015). Translation and validation of Hindi version of Oral Health Impact Profile-14. J. Indian Soc. Periodontol..

[9] Papagiannopoulou V., Oulis C.J., Papaioannou W., Antonogeorgos G., Yfantopoulos J. (2012). Validation of a Greek version of the oral health impact profile (OHIP-14) for use among adults. Health Qual. Life Outcomes.

[10] Gera A., Cattaneo P.M., Cornelis M.A. (2020). A Danish version of the oral health impact profile-14 (OHIP-14): Translation and cross-cultural adaptation. BMC Oral Health.

[11] Slusanschi O., Moraru R., Garneata L., Mircescu G., Cuculescu M., Preoteasa E. (2013). Validation of a Romanian version of the short form of the oral health impact profile (OHIP-14) for use in an urban adult population. Oral Health Prev. Dent..

[12] Skośkiewicz-Malinowska K., Kaczmarek U., Ziętek M., Malicka B. (2015). Validation of the Polish version of the oral health impact profile-14. Adv. Clin. Exp. Med..

[13] Montero-Martín J., Bravo-Pérez M., Albaladejo-Martínez A., Hernández-Martín L.A., Rosel-Gallardo E.M. (2009). Validation the Oral Health Impact Profile (OHIP-14sp) for adults in Spain. Med. Oral Patol. Oral Cir. Bucal.

[14] Warsi I., Younus A., Rasheed A., Ahmed J., Mahida H., Hashmi R., Qureshi A. (2018). Oral health-related quality of life in patients with upper gastrointestinal and hepatic disorders in Pakistan: Validation of the Oral Health Impact Profile-14 in the Urdu language. BDJ Open.

[15] Xin W.N., Ling J.Q. (2006). Validation of a Chinese version of the oral health impact profile-14. Zhonghua Kou Qiang Yi Xue Za Zhi.

[16] Hongxing L., List T., Nilsson I.M., Johansson A., Astrøm A.N. (2014). Validity and reliability of OIDP and OHIP-14: A survey of Chinese high school students. BMC Oral Health.

[17] Kushnir D., Zusman S.P., Robinson P.G. (2004). Validation of a Hebrew version of the Oral Health Impact Profile 14. J. Public Health Dent..

[18] León S., Bravo-Cavicchioli D., Correa-Beltrán G., Giacaman R.A. (2014). Validation of the Spanish version of the Oral Health Impact Profile (OHIP-14Sp) in elderly Chileans. BMC Oral Health.

[19] Castrejón-Pérez R.C., Borges-Yáñez S.A. (2012). Derivation of the short form of the Oral Health Impact Profile in Spanish (OHIP-EE-14). Gerodontology.

[20] Santucci D., Camilleri L., Kobayashi Y., Attard N. (2014). Development of a Maltese version of oral health-associated questionnaires: OHIP-14, GOHAI, and the Denture Satisfaction Questionnaire. Int. J. Prosthodont..

[21] Kenig N., Sotiroska Ivanoska K., Nikolovska J. (2023). Psychometric Properties of the 14 Items Oral Health Impact Profile Questionnaire Translated into the Macedonian Language. Acta Stomatol. Croat..

[22] Balci N., Alkan N., Gurgan C.A. (2017). Psychometric properties of a Turkish version of the oral health impact profile-14. Niger. J. Clin. Pract..

[23] Kuo H.C., Chen J.H., Wu J.H., Chou T.M., Yang Y.H. (2011). Application of the Oral Health Impact Profile (OHIP) among Taiwanese elderly. Qual. Life Res..

[24] Bae K.H., Kim H.D., Jung S.H., Park D.Y., Kim J.B., Paik D.I., Chung S.C. (2007). Validation of the Korean version of the oral health impact profile among the Korean elderly. Community Dent. Oral Epidemiol..

[25] Rimal J., Shrestha A. (2015). Validation of Nepalese Oral Health Impact Profile14 and Assessment of Its Impact in Patients with Oral Submucous Fibrosis in Nepal. J. Nepal Health Res. Counc..

[26] Ekanayake L., Perera I. (2003). Validation of a Sinhalese translation of the Oral Health Impact Profile-14 for use with older adults. Gerodontology.

[27] Larsson P., John M.T., Hakeberg M., Nilner K., List T. (2014). General population norms of the Swedish short forms of oral health impact profile. J. Oral Rehabil..

[28] Khalifa N., Allen P.F., Abu-bakr N.H., Abdel-Rahman M.E. (2013). Psychometric properties and performance of the Oral Health Impact Profile (OHIP-14s-ar) among Sudanese adults. J. Oral Sci..

[29] Lawal F.B., Taiwo J.O., Arowojolu M.O. (2014). How valid are the psychometric properties of the oral health impact profile-14 measure in adult dental patients in Ibadan, Nigeria?. Ethiop. J. Health Sci..

[30] Soe K.K., Gelbier S., Robinson P.G. (2004). Reliability and validity of two oral health related quality of life measures in Myanmar adolescents. Community Dent. Health.

[31] Stancić I., Sojić L.T., Jelenković A. (2009). Adaptation of Oral Health Impact Profile (OHIP-14) index for measuring impact of oral health on quality of life in elderly to Serbian language. Vojnosanit. Pregl..

[32] Rener-Sitar K., Petricević N., Celebić A., Marion L. (2008). Psychometric properties of Croatian and Slovenian short form of oral health impact profile questionnaires. Croat. Med. J..

[33] Kachkachishvili I.D. (2013). Georgian version of the “Oral Health Impact Profile”. Georgian Med. News.

[34] John M.T., Micheelis W., Biffar R. (2004). Reference values in oral health-related quality of life for the abbreviated version of the Oral Health Impact Profile. Schweiz. Monatsschr. Zahnmed..

[35] John M.T., Reißmann D.R., Feuerstahler L., Waller N., Baba K., Larsson P., Čelebić A., Szabo G., Rener-Sitar K. (2014). Factor analyses of the Oral Health Impact Profile—Overview and studied population. J. Prosthodont. Res..

[36] John M.T., Reissmann D.R., Feuerstahler L., Waller N., Baba K., Larsson P., Celebić A., Szabo G., Rener-Sitar K. (2014). Exploratory factor analysis of the Oral Health Impact Profile. J. Oral Rehabil..

[37] John M.T., Feuerstahler L., Waller N., Baba K., Larsson P., Celebić A., Kende D., Rener-Sitar K., Reissmann D.R. (2014). Confirmatory factor analysis of the Oral Health Impact Profile. J. Oral Rehabil..

[38] John M.T., Rener-Sitar K., Baba K., Čelebić A., Larsson P., Szabo G., Norton W.E., Reissmann D.R. (2016). Patterns of impaired oral health-related quality of life dimensions. J. Oral Rehabil..

[39] John M.T., Miglioretti D.L., LeResche L., Koepsell T.D., Hujoel P., Micheelis W. (2006). German short forms of the Oral Health Impact Profile. Community Dent. Oral Epidemiol..

[40] John M.T. (2022). Standardization of dental patient-reported outcomes measurement using OHIP-5—Validation of “recommendations for use and scoring of oral health impact profile versions”. J. Evid.-Based Dent. Pract..

[41] Omara M., Stamm T., Bekes K. (2023). Lessons learned from the first steps of implementing value-based oral health care: A case study from the Medical University of Vienna. J. Evid.-Based Dent. Pract..

[42] Reissmann D.R. (2021). Methodological considerations when measuring oral health-related quality of life. J. Oral Rehabil..

[43] Kenig N., Nikolovska J. (2012). Assessing the psychometric characteristics of the Macedonian version of the Oral Health Impact Profile questionnaire (OHIP-MAC49). Oral Health Dent. Manag..

[44] Elencevski S., Apostolska S., Stancic I., Persic S., Popovac A., Kovacic I., Celebic A. (2021). Psychometric properties of North Macedonian version of the Oral Health Impact Profile for Edentulous Subjects. Srp. Arh. Celok. Lek..

[45] Waller N., John M.T., Feuerstahler L., Baba K., Larsson P., Peršić S., Kende D., Reißmann D.R., Rener-Sitar K. (2016). A 7-day recall period for a clinical application of the oral health impact profile questionnaire. Clin. Oral Investig..

[46] Bland J.M., Altman D.G. (1999). Measuring agreement in method comparison studies. Stat. Methods Med. Res..

[47] Shrout P.E., Fleiss J.L. (1979). Intraclass correlations: Uses in assessing rater reliability. Psychol. Bull..

[48] Allen P.F., McMillan A.S., Locker D. (2001). An assessment of sensitivity to change of the Oral Health Impact Profile in a clinical trial. Community Dent. Oral Epidemiol..

[49] Norman G.R., Sloan J.A., Wyrwich K.W. (2003). Interpretation of changes in health-related quality of life: The remarkable universality of half a standard deviation. Med. Care.

[50] Naik A., John M.T., Kohli N., Self K., Flynn P. (2016). Validation of the English-language version of 5-item Oral Health Impact Profile. J. Prosthodont. Res..

[51] Lü H., He F.M. (2020). Reliability and validity of the Chinese version of the 5-item oral health impact profile. Hua Xi Kou Qiang Yi Xue Za Zhi.

[52] Alhajj M.N., Halboub E., Khalifa N., Amran A.G., Reissmann D.R., Abdullah A.G., Assad M., Al-Basmi A.A., Al-Ghabri F.A. (2018). Translation and validation of the Arabic version of the 5-item Oral health impact profile: OHIP5-Ar. Health Qual. Life Outcomes.

[53] Simancas-Pallares M., John M.T., Enstad C., Lenton P. (2020). The Spanish Language 5-Item Oral Health Impact Profile. Int. Dent. J..

[54] Nazeri A.M., Nakhaee N., Navabi N. (2020). Validation of an Ultrashort Persian Version of Oral Health Impact Profile (OHIP-5) Questionnaire. Pesqui. Bras. Odontopediatria Clin. Integr..

[55] León S., Correa-Beltrán G., De Marchi R.J., Giacaman R.A. (2017). Ultra-short version of the oral health impact profile in elderly Chileans. Geriatr. Gerontol. Int..

[56] Baba K., Inukai M., John M.T. (2008). Feasibility of oral health-related quality of life assessment in prosthodontic patients using abbreviated Oral Health Impact Profile questionnaires. J. Oral Rehabil..

[57] Nikolovska J., Korunoska-Stevkovska V., Mijoska A., Popovska L. (2018). Prosthodontics Status and Treatment Needs Among the Elderly in the Republic of Macedonia. Open Access Maced. J. Med. Sci..

[58] Stavreva N., Guguvcevchi L., Kapusevska B. (2015). Influence of the Ethnic Affiliation, Level of Education and Place of Living on Oral Health at Geriatric Population with Total and Partial Dentures in Republic of Macedonia. Prilozi.

[59] Bimbashi V., Celebić A., Islami A., Asllani-Hoxha F., Petricević N. (2012). Psychometric properties of the Albanian language version of the OHIP-ALB49 Questionnaire in the Republic of Kosovo. Coll. Antropol..

[60] Oweis Y., Ereifej N., Al-Asmar A., Nedal A. (2022). Factors Affecting Patient Satisfaction with Complete Dentures. Int. J. Dent..

[61] Casucci A., Cagidiaco E.F., Verniani G., Ferrari M., Borracchini A. (2025). Digital vs. conventional removable complete dentures: A retrospective study on clinical effectiveness and cost-efficiency in edentulous patients: Clinical effectiveness and cost-efficiency analysis of digital dentures. J. Dent..

[62] Nedeljković Đ., Milić Lemić A., Kuzmanović Pfićer J., Stančić I., Popovac A., Čelebić A. (2023). Subjective and Objective Assessment of Chewing Performance in Older Adults with Different Dental Occlusion. Med. Princ. Pract..

[63] Vozza I., Manzon L., Passarelli P.C., Pranno N., Poli O., Grippaudo C. (2021). The Effects of Wearing a Removable-Partial-Denture on the Bite Forces: A Cross-Sectional Study. Int. J. Environ. Res. Public Health.

[64] Kovacic I., Celebic A., Zlataric D.K., Petricevic N., Bukovic D., Bitanga P., Mikelic B., Tadin A., Mehulić K., Ognjenović M. (2010). Decreasing of residual alveolar ridge height in complete denture wearers. A five year follow up study.. Coll. Antropol..

[65] Sokolowski A., Huber S., Arefnia B., Pichler A., Lorenzoni M., Sokolowski A. (2025). The influence of prosthetic treatments and implant-supported prostheses on posterior mandibular ridge atrophy: A retrospective cohort study. BMC Oral Health.

[66] Kilić D., Peršić-Kiršić S., Čelebić A. (2024). Traumatic Ulceration of the Vestibular Mucous Membrane After Insertion of Four Mini-Implants in the Atrophied Mandible: A Case Report. Case Rep. Dent..

[67] Andrei O.C., Mărgărit R., Tănăsescu L.A., Dăguci L., Dăguci C. (2016). Prosthetic rehabilitation of complete edentulous patients with morphological changes induced by age and old ill fitted dentures. Rom. J. Morphol. Embryol..

[68] Topić J., Poljak-Guberina R., Persic-Kirsic S., Kovacic I., Petricevic N., Popovac A., Čelebić A. (2022). Adaptation to New Dentures and 5 Years of Clinical Use: A Comparison Between Complete Denture and Mini-implant Mandibular Overdenture Patients Based on Oral Health-Related Quality of Life (OHRQoL) and Orofacial Esthetics. Acta Stomatol. Croat..

[69] Vaddamanu S.K., Vyas R., Pati S.K., Thakkar R., Kumar A., Badiyani B.K. (2021). Effect of Food Colorants on Color of Denture Base Acrylic Resins. J. Pharm. Bioallied Sci..

[70] Al-Qarni F.D., Goodacre C.J., Kattadiyil M.T., Baba N.Z., Paravina R.D. (2020). Stainability of acrylic resin materials used in CAD-CAM and conventional complete dentures. J. Prosthet. Dent..

[71] Kutkut A., Bertoli E., Frazer R., Pinto-Sinai G., Fuentealba Hidalgo R., Studts J. (2018). A systematic review of studies comparing conventional complete denture and implant retained overdenture. J. Prosthodont. Res..

[72] Celebic A., Kovacic I., Petricevic N., Alhajj M.N., Topic J., Junakovic L., Persic-Kirsic S. (2024). Clinical Outcomes of Three Versus Four Mini-Implants Retaining Mandibular Overdenture: A 5-Year Randomized Clinical Trial. Medicina.

[73] Park J.H., Shin S.W., Lee J.Y. (2023). Mini-implant mandibular overdentures under a two-step immediate loading protocol: A 4-6-year retrospective study. Gerodontology.

[74] AlHelal A.A., Alzaid A.A., Almujel S.H., Alsaloum M., Alanazi K.K., Althubaitiy R.O., Al-Aali K.A. (2024). Clinical Peri-Implant Parameters and Marginal Bone Loss for Early Mandibular Implant Overdentures: A Follow-Up of 60 Months. Medicina.

[75] Jayasinghe R.M., Attygalla M., Fonseka M.C.N., Abeysundara S.P., Thilakumara I.P., Jayasinghe R.D. (2024). Single versus two dental implants retained mandibular over dentures: Comparison of function, patient satisfaction, oral health-related quality of life and success of treatment. BMC Res. Notes.

[76] Celebic A., Kovacic I., Petricevic N., Puljic D., Popovac A., Kirsic S.P. (2023). Mini-Implants Retaining Removable Partial Dentures in Subjects Without Posterior Teeth: A 5-Year Prospective Study Comparing the Maxilla and the Mandible. Medicina.

[77] Shandilya A., Behera S., Sahu G.K., Mallick R.R., Husain Z., Chauhan R. (2021). Comparative Evaluation of the Effectiveness of Rotary Instrumentation over Manual Instrumentation with Ultrasonic Irrigation on Incidence, Duration, and Intensity of Postendodontic Pain: An In Vivo Study. J. Pharm. Bioallied Sci..

[78] Ribeiro E.P., Emídio A.G., Zanin G.T., Melo E Silva V.F.F., Lopes M.B., Guiraldo R.D., Berger S.B. (2023). Dental aesthetic perception of patients submitted to activated charcoal-based bleaching agents: A randomized clinical trial. J. Dent..

[79] Mirza M.B., Almuteb A.B., Alsheddi A.T., Hashem Q., Abuelqomsan M.A., AlMokhatieb A., AlBader S., AlShehri A. (2025). Cross-Sectional Analysis of the Challenges Faced by Undergraduate Dental Students During Root Canal Treatment (RCT) and the Oral Health-Related Quality of Life in Patients After RCT. Medicina.

[80] Paulson D.R., Chanthavisouk P., John M.T., Feuerstahler L., Chen X., Ingleshwar A. (2024). Linking patient-reported oral and general health-related quality of life. PeerJ.

[81] Solanke C., John M.T., Ebel M., Altner S., Bekes K. (2024). OHIP-5 for school-aged children. J. Evid.-Based Dent. Pract..

[82] Bertl K., Tsakos G., Pandis N., Bogren A., Burisch J., Stavropoulos A. (2023). Health-related quality of life aspects of the ‘Periodontitis prevalence in ulcerative colitis and Crohn’s disease’ (PPCC) cohort. J. Clin. Periodontol..

[83] Bekes K., Kuhr K., Ohm C., Frenzel Baudisch N., Jordan A.R. (2023). Does orthodontic treatment need have an impact on oral health-related quality of life?. J. Orofac. Orthop..

[84] Bida F.C., Agop-Forna D., Bulancea B.P., Balcoș C., Forna N.C. (2022). An Observational Study on Oral Health and Quality of Life for RPD Wearers in the N-E Region of Romania. Medicina.

[85] Ye Y., Wu J., Dai Y., Tan Y., You Y., Tan J. (2024). Psychological problems and their impact on oral mucosal disease patients’ quality of life: A cross-sectional study in the Chinese population. Heliyon.

[86] Agnese C.C.D., Schöffer C., Kantorski K.Z., Zanatta F.B., Susin C., Antoniazzi R.P. (2025). Periodontitis and Oral Health-Related Quality of Life: A Systematic Review and Meta-Analysis. J. Clin. Periodontol..

[87] AlSaggaf A.U., Alqutub A., Almasri Z., Khalifah F., Khuzaee F., Aljuaid A., Bukhari O., Marghalani A.A. (2024). Oral Health-Related Quality of Life Improvement After Treatment with Fixed and Removable Dental Prostheses. Cureus.

